# Poly-Amino-β-Cyclodextrin Microparticles for the Reduction of Xenobiotics and Emerging Contaminants, Including Pharmaceuticals, from the Natural Environment

**DOI:** 10.3390/ma17225424

**Published:** 2024-11-06

**Authors:** Wojciech Ciesielski, Damian Kulawik, Beata Girek, Kinga Kozieł-Trąbska, Iwona Zawierucha, Tomasz Girek

**Affiliations:** Faculty of Science and Technology, Jan Dlugosz University in Czestochowa, Armii Krajowej Ave. 13/15, 42-201 Czestochowa, Poland; d.kulawik@ujd.edu.pl (D.K.); b.girek@ujd.edu.pl (B.G.); kingakoziel123@gmail.com (K.K.-T.); i.zawierucha@ujd.edu.pl (I.Z.); t.girek@ujd.edu.pl (T.G.)

**Keywords:** β-cyclodextrin polymer, hydrogel composite material, amino-β-cyclodextrin, adsorption of hormones, adsorption of non-steroidal anti-inflammatory drugs

## Abstract

The contamination of the natural environment by xenobiotics and emerging contaminants, including pharmaceuticals, poses significant risks to ecosystems and human health. Among these contaminants, hormones and pharmaceutical compounds are particularly concerning due to their persistence and potential biological effects even at low concentrations. In this study, we investigated the efficacy of poly-amino-β-cyclodextrin (PA-β-CD) microparticles in adsorbing and reducing specific xenobiotics and pharmaceuticals from aqueous solutions. Our research focused on four contaminants: two hormones, testosterone and progesterone, and two pharmaceutical drugs, diclofenac and carbamazepine. High-performance liquid chromatography (HPLC) was employed to quantify the adsorption capacity and efficiency of PA-β-CD microparticles.

## 1. Introduction

Hydrogels (HG) are three-dimensional polymer networks capable of absorbing large amounts of water. Hydrogel composites (HGC) are chemically and physically stable, possessing a flexible polymer network that can be reused multiple times and applied in various fields. These flexible materials retain the ability to swell and hold a substantial amount of water within their structure, without dissolving in water [1,2]. One of the main development directions for these materials is environmental remediation, where hydrogel composites have shown high efficiency in adsorbing inorganic pollutants, including heavy metals, and organic contaminants such as pharmaceuticals and pesticides [3,4].

Cyclodextrins (CDs) are cyclic oligomers derived from the enzymatic degradation of starch. CDs consist of six, seven, or eight glucopyranosyl units (called α-, β-, and γ-CD, respectively), which are connected by α-1,4 bonds. CDs are homogeneous crystalline substances, water-soluble, and possess a ring-shaped structure that narrows on one side and widens on the other [5,6]. Due to the specific arrangement of hydroxyl groups on the edges of the ring, cyclodextrins can be easily modified, expanding their applications in various fields such as chemosensors, artificial enzymes, and drug carriers [7]. The use of cyclodextrins as precursors for the creation of derivatives and polymers is justified by their biodegradability and biocompatibility with human tissues [8].

One of the main challenges in modifying hydrogels and cyclodextrins is developing a fully regenerable, reusable material that retains its stability and effectiveness [9,10,11]. The polymerization of cyclodextrins enhances their stability and reduces solubility, making them more useful in environmental and medical applications [12]. A new class of polymers based on β-cyclodextrin cross-linked with dianhydrides introduces anionic groups into the polymer network, significantly increasing their potential in forming hydrogels that can interact with both polycations and polyanions [13,14]. Due to this polyelectrolyte property, cyclodextrin polymers can form complexes with therapeutic substances and facilitate transport through biomembranes [15].

The presented β-cyclodextrin polymer, modified with an amino group, exhibits both polyampholytic properties and higher solubility compared to unmodified polymers [16,17]. With the ability to form porous microspheres, cyclodextrins and their derivatives are widely used as controlled release systems for substances, including drugs and fertilizers [18]. β-cyclodextrin polymers can be successfully employed as drug delivery systems that encapsulate and gradually release active substances, which has broad applications in pharmacology and agriculture [19,20,21,22,23].

Studies have shown that PA-β-CD microcapsules are effective in adsorbing a wide range of compounds due to the synergistic action of the cyclodextrin cavity and amino groups. The cyclodextrin cavity creates an optimal hydrophobic environment for the encapsulation of organic compounds, while the amino groups enhance interactions through hydrogen bonding and electrostatic forces. This dual functionality is advantageous for the adsorption of diverse pollutants [6,7,24].

In studies on the adsorption of hormones and non-steroidal anti-inflammatory drugs (NSAIDs), the effect of pH on adsorption efficiency was carefully analyzed. Optimal pH conditions were determined for both PA-β-CD and sodium alginate hydrogels (SA), and maximum adsorption times were assessed.

The conducted studies emphasize the potential of PA-β-CD as a highly effective material for a wide range of adsorption applications, particularly in varying pH conditions, while also showcasing the durability and versatility of both PA-β-CD and SA systems in the adsorption of hormones and NSAIDs [25].

## 2. Materials and Methods

β-Cyclodextrin (β-CD), N, N-dimethyl formamide (DMF), and sodium hydride were purchased from Sigma-Aldrich, St. Louis, MO, USA. DMF was distilled under a vacuum. The dried DMF was stored in a dark bottle over calcium hydride. Sodium hydride (60% in oil) was dried with hexane. Sodium alginate with low viscosity was purchased from Buchi, Switzerland, and pyromellitic dianhydride (PA) was purchased from Alfa Aesar, Ward Hill, MA, USA. Acetone, acetic acid, and hexane were purchased from Chempur, Piekary Slaskie, Poland. Water, HPLC-grade; methanol, HPLC-grade; and acetonitrile, HPLC grade, were purchased from Thermo Scientific Chemicals, Waltham, MA, USA. Acetonitrile, HPLC-grade, was purchased from Thermo Scientific Chemicals, USA. Testosterone, analytical standard; progesterone, analytical standard; and carbamazepine and diclofenac sodium salt, analytical standard, were purchased from Supelco^®^ Analytical Products, Darmstadt, Germany. Mono-6-azido-6-deoxy-β-cyclodextrin (NβCD), Mono-6-amino-6-deoxy-β-cyclodextrin (AβCD), and blocking the amine group by BOC (BOCAβCD) were synthesized according to the procedure in [26]. PA-β-CD microparticles were synthesized through a modified polymerization process involving β-cyclodextrin and amino-functional monomers. Microparticles were formed using the obtained PA-β-CD polymer and sodium alginate solution via the vibration technique on a Buchi B-395 Pro encapsulator, Uster, Switzerland [27].

### 2.1. Encapsulation Process

The next step in obtaining the substance-adsorbing system was the production of hydrogel microspheres through the encapsulation of PA-β-CD with sodium alginate. Microparticles were formed using the vibration technique on a Buchi B-395 Pro encapsulator. The 1.5% solution of alginate and a 0.5% aqueous solution of the resulting polymer (60 mL) were prepared. The parameters of the encapsulation are given in Table 1. An amount of 1 mM CaCl_2_ cross-linker solution was made. A solution of the polymer and alginate was added to the curing solution and placed on a magnetic stirrer.

**Figure 1:**
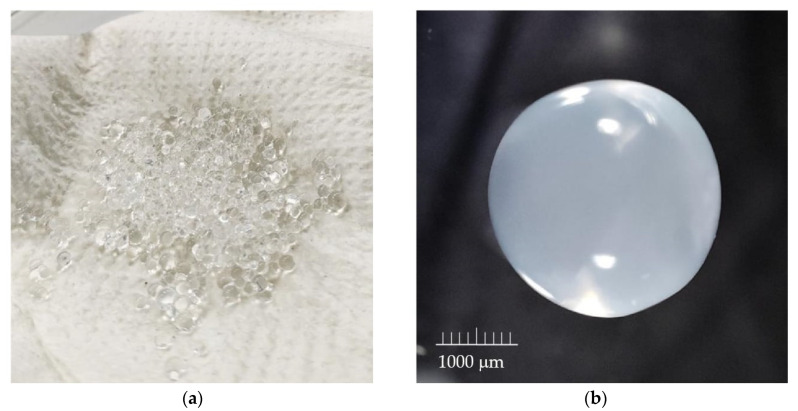
(**a**) Pictures of SAPAβCD microparticles (sodium alginate poly-amino-β-cyclodextrin) directly from the encapsulator and (**b**) at 40× zoom.

In all the studies presented below, spheres from a nozzle with a diameter of 1000 µm were used. The sphere diameter is approximately 2 mm (Figure 1).

### 2.2. Adsorption Experiment

The adsorption experiments were conducted using water/methanol (H_2_O/MeOH) solutions for testosterone and progesterone and water/acetonitrile (H_2_O/MeCN) solutions for diclofenac and carbamazepine. Initial concentrations of each contaminant were standardized, and SAPAβCD microparticles were added to the solutions (Table 2).

Polymer samples in the form of hydrogel spheres with a diameter of approximately 2 mm and a mass of 1 g were weighed and placed in 1.5 mL Eppendorf tubes.

In the first part of the study, aimed at determining the optimal adsorption environment, the polymer samples were immersed in an aqueous solution of the tested compound. The pH of the solution was adjusted using a pH meter and then maintained at a constant level by adding NaOH or HCl. The samples were left under these conditions for 24 h (Table 3).

In the second part of the study, aimed at determining the maximum adsorption time, the polymer samples were immersed in an aqueous solution of the tested compound for a specified time, which was gradually increased in subsequent experiments (Table 4).

In both parts of the study, the adsorption processes were monitored to determine the kinetics and efficiency of the tested compound’s uptake by the polymer hydrogel spheres.

After the predetermined time had elapsed, the solution was decanted, filtered through 0.22 µm syringe filters, transferred to chromatography vials, placed in the autosampler of the chromatograph, and analyzed.

### 2.3. High-Perfprmance Liquid Chromatografy (HPLC) Methods

The study of maximum organic substance adsorption was conducted using the Agilent 1260 Infinity II LC System high-performance liquid chromatograph, equipped with an Ultivo Triple Quadrupole LC/MS mass spectrometer with electrospray ionization (ESI) (Agilent Technologies, Santa Clara, CA, USA). The conditions for each measurement are presented in Table 5. An InfinityLab Poroshell 120 EC-C18 column, 4.6 × 100 mm, with a pore size of 4 µm (Agilent Technologies, Santa Clara, CA, USA), was used. Standard solutions of the samples were prepared using analytical standards of carbamazepine [Sigma-Aldrich, St. Louiis, MO, USA C4024-5G ≥98%, powder, antiepileptic agent]; diclofenac sodium salt (Sigma-Aldrich D6899-10G ≥98%, powder]; progesterone [Sigma-Aldrich P8783-5G ≥99% powder, BioReagent, suitable for cell culture]; and testosterone [Sigma-Aldrich 86500-5G purity, ≥99.0% powder]. The optimized MRM (multiple reaction monitoring) parameters for the analyzed compounds are represented in Table 6.

Chromatograms were analyzed using Agilent Mass Hunter Qualitative Analysis 10.0 and QQQ Quantitative Analysis (Quant-My-Way) v10.2 software.

### 2.4. Preparation of HPLC Calibration Curves

Standard substance samples of 0.1 g were prepared by weighing them on a balance with an accuracy of 0.001 g. The weighed samples were then quantitatively transferred to a 1000 mL volumetric flask and filled to the mark with the appropriate solvent. For testosterone, progesterone, and carbamazepine, the solvent was methanol/water (70:30), while for diclofenac, it was acetonitrile/water (75:25). The resulting solutions, with a concentration of 100 ppm, were used to prepare dilutions. Measurements were conducted five times to ensure repeatability and accuracy of the results.

**Figure 2:**
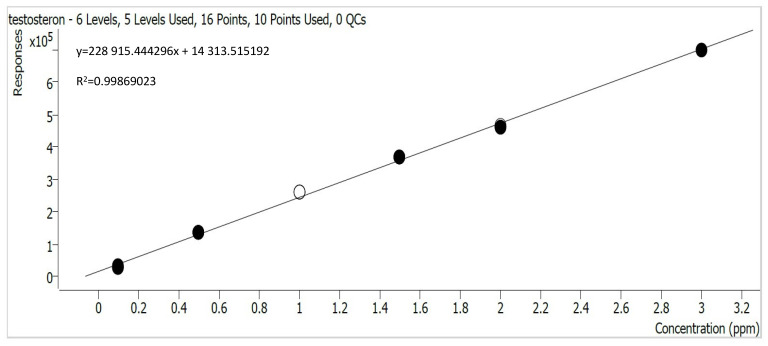
Testosterone calibration curve.

**Figure 3:**
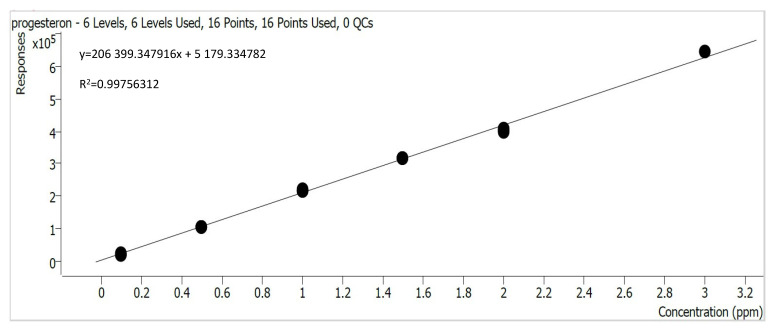
Progesterone calibration curve.

**Figure 4:**
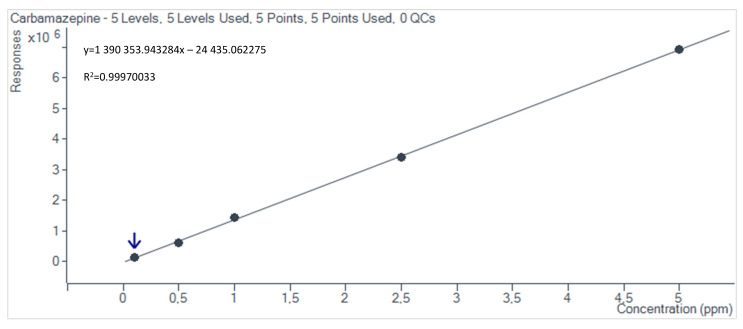
Carbamazepine calibration curve.

**Figure 5:**
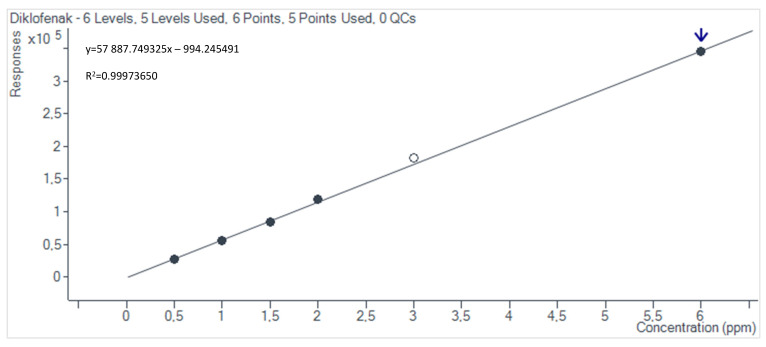
Diclofenac calibration curve.

The obtained calibration curves, determined using the Mass Hunter Quantitative Analysis (Quant-MyWay) software, are presented in Figure 2, Figure 3, Figure 4 and Figure 5.

## 3. Results and Discussion

The high adsorption capacities and efficiencies observed for PA-β-CD microparticles can be attributed to the combined effects of the cyclodextrin cavity and the amino functional groups. The cyclodextrin cavity provides a hydrophobic environment suitable for encapsulating organic molecules, while the amino groups enhance interaction through hydrogen bonding and electrostatic forces. This dual functionality makes PA-β-CD microparticles particularly effective for adsorbing a wide range of contaminants.

The analysis of the adsorption of hormones and drugs from the non-steroidal anti-inflammatory drug (NSAID) group was conducted over a wide pH range to study the impact of this parameter on the efficiency of the adsorption process. Additionally, the analysis was extended to determine the maximum adsorption time under selected conditions.

To investigate the effect of pH on adsorption, a series of experiments was conducted at different pH values. Samples showing adsorption at maximum pH values were identified. These selected samples were then further analyzed to determine the maximum adsorption time, allowing for the identification of optimal conditions for this process.

The research process involved systematically varying the pH values and monitoring the adsorption efficiency of hormones and NSAIDs. Selected samples, where adsorption occurred at maximum pH values, were further analyzed to determine the maximum adsorption time. These studies allowed for an understanding of the effect of pH on the adsorption process and for the identification of optimal conditions for this process in the context of hormones and NSAIDs.

To demonstrate that the PA-β-CD polymer (PAβCD) is responsible for the adsorption of the tested compounds, comparative studies were conducted using hydrogel microcapsules (SA). The hydrogel microcapsules were synthesized using a simple sodium alginate cross-linking method.

### 3.1. Testosterone and Progesterone Adsorption

The analysis of hormone adsorption was carried out using a liquid chromatograph equipped with a mass detector. In the first stage of the study, the optimal pH conditions for the adsorption of hormones by microcapsules with the PA-β-CD polymer and hydrogels cross-linked with sodium alginate (SA) were determined. Next, for a polymer sample weighing 1 g, the maximum adsorption value was established as a function of the adsorption time.

**Figure 6:**
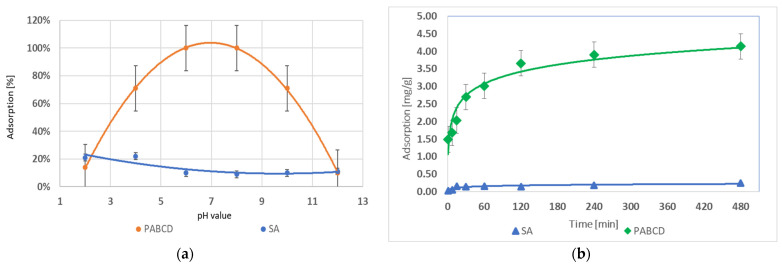
(**a**) Testosterone adsorption in the range pH 2–12 and (**b**) testosterone adsorption over time.

In the studies on testosterone absorption by microcapsules with the PA-β-CD polymer and hydrogels cross-linked with sodium alginate (SA), it was observed that the highest adsorption percentage for PA-β-CD was 100% at pH 6 and 8. For SA, the best result was achieved at pH 4, where the adsorption was 22%. The optimal pH range for PA-β-CD is 5–9, while pH does not affect the adsorption efficiency for SA. Both strongly acidic and basic environments negatively impacted the adsorption capacity of PA-β-CD (Figure 6a).

The analysis of the maximum testosterone adsorption time showed that a hydrogel system containing 1 g of polymer could adsorb a maximum of 0.25 mg/g of testosterone for SA and 4.14 mg/g for PA-β-CD. The results are presented in the graph (Figure 6b).

**Figure 7:**
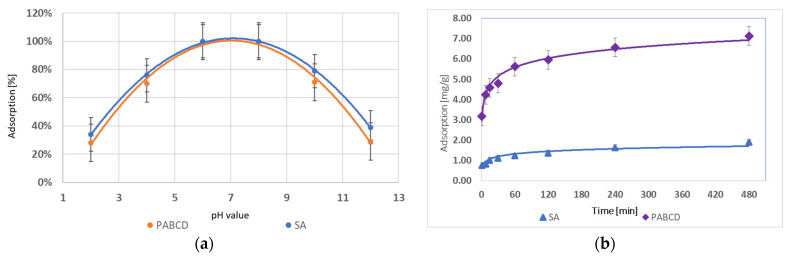
(**a**) Progesterone adsorption in the range pH 2–12 and (**b**) progesterone adsorption over time.

In the studies on progesterone adsorption by microcapsules with the PA-β-CD polymer and hydrogels cross-linked with sodium alginate (SA), it was found that the highest absorption percentage for both systems was 100% at pH 6 and 8. The optimal pH range for both PA-β-CD and SA is 5–9. Both strongly acidic and basic environments negatively impacted the adsorption capacity of both PA-β-CD and SA. The results are presented in the graph (Figure 7a).

The analysis of the maximum progesterone adsorption time showed that a hydrogel system containing 1 g of polymer could adsorb a maximum of 1.90 mg/g of testosterone for SA and 7.13 mg/g for PA-β-CD. The results are presented in the graph (Figure 7b).

### 3.2. Non-Steroidal Anti-Inflammatory Drug Adsorption

The analyses of selected NSAID adsorption were carried out using a liquid chromatograph equipped with a mass detector. In the first stage of the study, the optimal pH conditions for the adsorption of NSAIDs by microcapsules with the PA-β-CD polymer and hydrogels cross-linked with sodium alginate (SA) were determined. Next, for a polymer sample weighing 1 g, the maximum adsorption value was established as a function of the adsorption time similarly to the hormone investigation.

**Figure 8:**
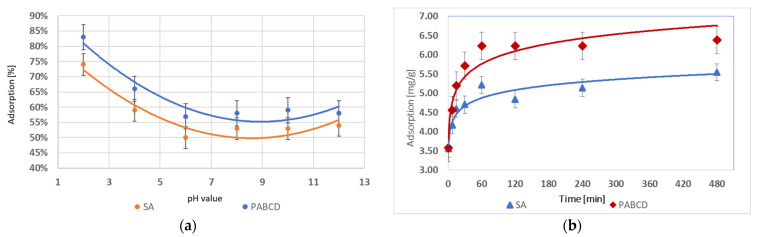
(**a**) Diclofenac adsorption in the range pH 2–12 and (**b**) diclofenac adsorption over time.

In the studies on the adsorption of diclofenac by microcapsules with the PA-β-CD polymer and hydrogels cross-linked with sodium alginate (SA), it was found that the highest adsorption percentage for PA-β-CD was 83% at pH 2, and for SA, it was 74% at pH 2. The optimal pH values for PA-β-CD and SA are 2 and 12, respectively. Both strongly acidic and basic environments favored increased adsorption capacity for both systems. The results are presented in the graph (Figure 8a).

The pH 2 condition was selected for the adsorption time analysis. The analysis of the maximum adsorption time for diclofenac showed that the hydrogel system containing 1 g of polymer could adsorb up to 5.54 mg/g of diclofenac for SA and 6.38 mg/g for PA-β-CD. The results are presented in the graph (Figure 8b).

**Figure 9:**
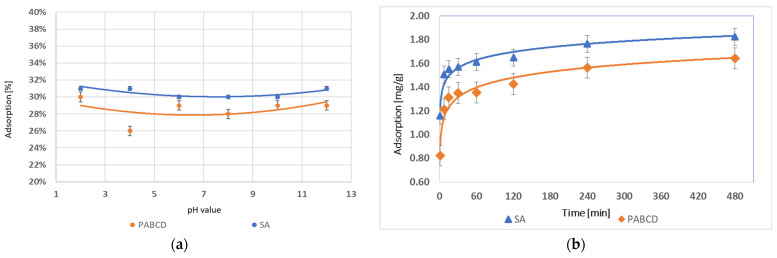
(**a**) Carbamazepine adsorption in the range pH 2–12 and (**b**) carbamazepine adsorption over time.

In the studies on carbamazepine adsorption by microcapsules with the PA-β-CD polymer and hydrogels cross-linked with sodium alginate (SA), it was found that the highest adsorption percentage for PA-β-CD was 30% at pH 2, 4, and 12, and for SA, it was 31% at pH 2. The differences in adsorption across a wide pH range were minimal, indicating that pH has no significant effect on the carbamazepine adsorption capacity of either system. The results are presented in the graph (Figure 9a).

In the analysis of the maximum adsorption time of carbamazepine by the hydrogel system containing 1 g of polymer, it was found that the maximum amount of carbamazepine adsorbed was 1.83 mg/g for SA and 1.64 mg/g for PA-β-CD. The differences in adsorption were minimal, suggesting that the addition of the PA-β-CD polymer does not have a significant impact on the carbamazepine adsorption capacity of either system. The observed minor differences in adsorption indicate a similar efficiency of both systems in adsorbing carbamazepine, which may result from the lack of a significant effect of the PA-β-CD polymer on this process. The results are presented in the graph (Figure 9b).

## 4. Conclusions

Our study highlights the potential of poly-amino-β-cyclodextrin microparticles as an efficient adsorbent for the reduction of xenobiotics and emerging contaminants, including pharmaceuticals, from aqueous environments. The significant adsorption capacities for testosterone, progesterone, diclofenac, and carbamazepine demonstrate the versatility and effectiveness of PA-β-CD microparticles.

This research demonstrates the effectiveness of PA-β-CD microparticles in adsorbing a diverse range of compounds due to the synergistic effects of the cyclodextrin cavity and amino functional groups. The cyclodextrin cavity provides an optimal hydrophobic environment for encapsulating organic molecules, while the amino groups enhance interactions through hydrogen bonding and electrostatic forces. This dual functionality proves advantageous in adsorbing various contaminants.

For hormone and NSAID adsorption studies, the impact of pH on adsorption efficiency was thoroughly investigated. Optimal pH conditions were determined for both PA-β-CD and sodium alginate (SA) hydrogels, and the maximum adsorption times were assessed. The results reveal that PA-β-CD demonstrates superior adsorption capabilities compared to SA across different conditions. Specifically, PA-β-CD achieved complete adsorption of testosterone and progesterone at pH 6 and 8, whereas SA showed lower adsorption efficiencies at these pH levels. For NSAIDs like diclofenac, PA-β-CD also performed better than SA in adsorption percentages, particularly at pH 2, with increased adsorption capacities observed in both strongly acidic and basic environments.

In contrast, the adsorption of carbamazepine showed minimal pH dependency, with both PA-β-CD and SA exhibiting similar adsorption efficiencies. The observed differences in adsorption capacities for carbamazepine between the two systems were negligible, indicating that the PA-β-CD polymer does not significantly enhance carbamazepine adsorption compared to SA.

The effect of pH on the absorption rate of the drugs tested depends on several key factors.

The specific chemical structure of hormones and NSAIDs, including the presence of acidic or basic functional groups, influences how they interact with the surrounding environment at different pH levels. This can lead to changes in their adsorption characteristics.

Moreover, the pH may influence the interactions between the drugs and the adsorbent materials such as poly-amino-β-cyclodextrin. For instance, changes in pH can affect the charge and polarity of the adsorbents, altering their binding affinity for the drugs.

Also, the pH of the environment can affect not only the drugs but also the properties of the polymeric materials used for encapsulation or adsorption. This can influence how effectively the drugs are captured and released.

The pH can also influence the hydrophobic or hydrophilic nature of both the drugs and the adsorbents, impacting how readily the drugs can be adsorbed or adsorbed under varying conditions.

Overall, this study underscores the potential of PA-β-CD as a highly effective material for a wide range of adsorption applications, particularly in varying pH environments, while highlighting the robustness and versatility of both PA-β-CD and SA systems in hormone and NSAID adsorption.

## Figures and Tables

**Table 1:** The parameters of the encapsulation.

Nozzle Diameter (µm)	Concentration of Sodium Alginate [%]	Flow Rate [mL/min]	Frequency [Hz]	Voltage [V]	Amplitude
1000	2	30	100	500	0,4
450	1.5	9	300	500	0,5
80	1	1	2000	500	0,6

**Table 2:** Initial concentrations of compounds.

	Testosterone	Progesterone	Carbamazepine	Diclofenac
Initial concentrations [ppm]	2.3829	4.5496	3.5364	5.2319
Solvent	70% MeOH/30% H_2_O	70% MeOH/30% H_2_O	70% MeCN/30% H_2_O	75% MeCN/25% H_2_O

**Table 3:** pH of the solutions for all compounds.

pH Values Used for Analysis						
pH	2	3	4	5	10	12

**Table 4:** Adsorption time for all compounds.

Adsorption Time									
Time [min]	1	7.5	15	30	60	120	240	360	480

**Table 5:** Chromatographic conditions.

Compound		Eluent	Flow Rate [ml/min]	Injection Volume [µL]	Column Temp. [°C]
Testosterone	A	H_2_O + 20 mM AF * + 0.1% FA **	0.7	5	50
	B	MeOH + 0.1% FA			
Progesterone	A	H_2_O + 20 mM AF + 0.1% FA	0.7	5	50
	B	MeOH + 0.1% FA			
Carbamazepine	A	H_2_O + 5 mM AF + 0.1% FA	0.8	5	40
	B	90% MeCN + 5 mM AF + 0.1% FA			
Diclofenac	A	H_2_O + 5 mM AF + 0.1% FA	0.8	5	40
	B	MeCN + 5 mM AF + 0.1% FA			

* AF—ammonium formate; ** FA—formic acid.

**Table 6:** Optimized MRM parameters for analyzed compounds.

Compound	RT (min)	Molecular Weight	Polarity	MRM Transition (*m*/*z*)	Collision Energy (V)
Testosterone	5.86	288.4	Positive	289.3 > 97.1	23
Progesterone	7.44	314.5	Positive	315.2 > 97.1	25
Carbamazepine	2.13	236.3	Positive	237.1 > 194.1; 237.1 >194.1	16; 32
Diclofenac	2.32	296.2	Positive	296 > 250; 296 > 215	8; 12

## Data Availability

The original contributions presented in the study are included in the article, further inquiries can be directed to the corresponding author.

## References

[1] Zhao W., Jin X., Cong Y., Liu Y., Fu J. (2013). Degradable natural polymer hydrogels for articular cartilage tissue engineering. J. Chem. Technol. Biotechnol..

[2] Shetye S.P., Godbole D.A.M., Bhilegaokar D.S., Gajare P.S. (2015). Hydrogels: Introduction, Preparation, Characterization and Applications. Hum. J..

[3] Gao X., Guo C., Hao J., Zhao Z., Long H., Li M. (2020). Adsorption of heavy metal ions by sodium alginate based adsorbent-a review and new perspectives. Int. J. Biol. Macromol..

[4] Shao Z.-J., Huang X.-L., Yang F., Zhao W.-F., Zhou X.-Z., Zhao C.-S. (2018). Engineering sodium alginate-based cross-linked beads with high removal ability of toxic metal ions and cationic dyes. Carbohydr. Polym..

[5] Szejtli J. (1998). Introduction and general overview of cyclodextrin chemistry. Chem. Rev..

[6] Dodziuk H. (2006). Cyclodextrins and Their Complexes: Chemistry, Analytical Methods, Applications.

[7] Sliwa W., Girek T. (2017). Cyclodextrins: Properties and Applications.

[8] Davis M.E., Brewster M.E. (2004). Cyclodextrin-based pharmaceutics: Past, present and future. Nat. Rev. Drug Discov..

[9] Zhang Y., Li X., Zhong N., Huang Y., He K., Ye X. (2019). Injectable in situ dual-crosslinking hyaluronic acid and sodium alginate based hydrogels for drug release. J. Biomater. Sci. Polym. Ed..

[10] Batool S.R., Nazeer M.A., Ekinci D., Sahin A., Kizilel S. (2020). Multifunctional alginate-based hydrogel with reversible crosslinking for controlled therapeutics delivery. Int. J. Biol. Macromol..

[11] Siboro S.A.P., Anugrah D.S.B., Ramesh K., Park S.-H., Kim H.-R., Lim K.T. (2021). Tunable porosity of covalently crosslinked alginate-based hydrogels and its significance in drug release behavior. Carbohydr. Polym..

[12] Pellicer J.A., Rodríguez-López M.I., Fortea M.I., Lucas-Abellán C., Mercader-Ros M.T., López-Miranda S., Gómez-López V.M., Semeraro P., Cosma P., Fini P. (2019). Adsorption properties of β-and hydroxypropyl-β-cyclodextrins cross-linked with epichlorohydrin in aqueous solution. A sustainable recycling strategy in textile dyeing process. Polymers.

[13] Kobayashi Y., Nakamitsu Y., Zheng Y., Takashima Y., Yamaguchi H., Harada A. (2019). Preparation of cyclodextrin-based porous polymeric membrane by bulk polymerization of ethyl acrylate in the presence of cyclodextrin. Polymer.

[14] Rojas-Aguirre Y., Torres-Mena M.A., López-Méndez L.J., Alcaraz-Estrada S.L., Guadarrama P., Urucha-Ortíz J.M. (2019). PEGylated β-cyclodextrins: Click synthesis and in vitro biological insights. Carbohydr. Polym..

[15] Malanga M., Seggio M., Kirejev V., Fraix A., Di Bari I., Fenyvesi E., Ericson M.B., Sortino S. (2019). A phototherapeutic fluorescent β-cyclodextrin branched polymer delivering nitric oxide. Biomater. Sci..

[16] Thakur S., Verma A., Raizada P., Gunduz O., Janas D., Alsanie W.F., Scarpa F., Thakur V.K. (2022). Bentonite-based sodium alginate/dextrin cross-linked poly (acrylic acid) hydrogel nanohybrids for facile removal of paraquat herbicide from aqueous solutions. Chemosphere.

[17] Girek T., Koziel K., Girek B., Ciesielski W. (2020). CD oxyanions as a tool for synthesis of highly anionic cyclodextrin polymers. Polymers.

[18] Ciesielska A., Ciesielski W., Girek B., Girek T., Koziel K., Kulawik D., Lagiewka J. (2020). Biomedical application of cyclodextrin polymers cross-linked via dianhydrides of carboxylic acids. Appl. Sci..

[19] Trotta F., Zanetti M., Cavalli R. (2012). Cyclodextrin-based nanosponges as drug carriers. Beilstein J. Org. Chem..

[20] Rafati N., Zarrabi A., Caldera F., Trotta F., Ghias N. (2019). Pyromellitic dianhydride crosslinked cyclodextrin nanosponges for curcumin controlled release; formulation, physicochemical characterization and cytotoxicity investigations. J. Microencapsul..

[21] Cecone C., Zanetti M., Anceschi A., Caldera F., Trotta F., Bracco P. (2019). Microfibers of microporous carbon obtained from the pyrolysis of electrospun β-cyclodextrin/pyromellitic dianhydride nanosponges. Polym. Degrad. Stab..

[22] Concheiro A., Alvarez-Lorenzo C. (2013). Chemically cross-linked and grafted cyclodextrin hydrogels: From nanostructures to drug-eluting medical devices. Adv. Drug Deliv. Rev..

[23] Sharaf S., El-Naggar M.E. (2019). Wound dressing properties of cationized cotton fabric treated with carrageenan/cyclodextrin hydrogel loaded with honey bee propolis extract. Int. J. Biol. Macromol..

[24] Boczar D., Michalska K. (2022). Cyclodextrin Inclusion Complexes with Antibiotics and Antibacterial Agents as Drug-Delivery Systems-A Pharmaceutical Perspective. Pharmaceutics.

[25] Folch-Cano C., Yazdani-Pedram M., Olea-Azar C. (2014). Inclusion and functionalization of polymers with cyclodextrins: Current applications and future prospects. Molecules.

[26] Kozieł K., Łagiewka J., Girek B., Folentarska A., Girek T., Ciesielski W. (2021). Synthesis of New Amino-β-Cyclodextrin Polymer, Cross-Linked with Pyromellitic Dianhydride and Their Use for the Synthesis of Polymeric Cyclodextrin Based Nanoparticles. Polymers.

[27] Kozieł-Trąbska K., Żarska S., Girek T., Ciesielski W. (2023). Characterization of New Polymer Material of Amino-β-Cyclodextrin and Sodium Alginate for Environmental Purposes. Membranes.

